# Renal Impairment with Sublethal Tubular Cell Injury in a Chronic Liver Disease Mouse Model

**DOI:** 10.1371/journal.pone.0146871

**Published:** 2016-01-11

**Authors:** Tokiko Ishida, Hirokazu Kotani, Masashi Miyao, Chihiro Kawai, Leila Jemail, Hitoshi Abiru, Keiji Tamaki

**Affiliations:** Department of Forensic Medicine, Kyoto University Graduate School of Medicine, Kyoto, Japan; University of Louisville, UNITED STATES

## Abstract

The pathogenesis of renal impairment in chronic liver diseases (CLDs) has been primarily studied in the advanced stages of hepatic injury. Meanwhile, the pathology of renal impairment in the early phase of CLDs is poorly understood, and animal models to elucidate its mechanisms are needed. Thus, we investigated whether an existing mouse model of CLD induced by 3,5-diethoxycarbonyl-1,4-dihydrocollidine (DDC) shows renal impairment in the early phase. Renal injury markers, renal histology (including immunohistochemistry for tubular injury markers and transmission electron microscopy), autophagy, and oxidative stress were studied longitudinally in DDC- and standard diet–fed BALB/c mice. Slight but significant renal dysfunction was evident in DDC-fed mice from the early phase. Meanwhile, histological examinations of the kidneys with routine light microscopy did not show definitive morphological findings, and electron microscopic analyses were required to detect limited injuries such as loss of brush border microvilli and mitochondrial deformities. Limited injuries have been recently designated as *sublethal tubular cell injury*. As humans with renal impairment, either with or without CLD, often show almost normal tubules, sublethal injury has been of particular interest. In this study, the injuries were associated with mitochondrial aberrations and oxidative stress, a possible mechanism for sublethal injury. Intriguingly, two defense mechanisms were associated with this injury that prevent it from progressing to apparent cell death: autophagy and single-cell extrusion with regeneration. Furthermore, the renal impairment of this model progressed to chronic kidney disease with interstitial fibrosis after long-term DDC feeding. These findings indicated that DDC induces renal impairment with sublethal tubular cell injury from the early phase, leading to chronic kidney disease. Importantly, this CLD mouse model could be useful for studying the pathophysiological mechanisms of sublethal tubular cell injury.

## Introduction

Acute kidney injury (AKI) is a common complication of chronic liver diseases (CLDs), and the prevalence is approximately 20% in patients with cirrhosis [1, 2]. The pathogenesis of renal impairment in CLD has been primarily studied in the advanced stages of hepatic injury both in animal models and clinical studies [2, 3]. These studies revealed that hepatorenal syndrome and bile nephrosis are two major pathophysiological mechanisms of AKI in advanced hepatic failure [2–5]. Meanwhile, the definitive pathological features of renal impairment in the early phase of CLDs is poorly understood in part because of the lack of animal models. Animal models to elucidate its mechanisms could lead to development of novel therapeutic and protective strategies against renal impairment in patients with CLDs. Thus, this study focuses on renal impairment in the early phase of CLD induced in mice by 3,5-diethoxycarbonyl-1,4-dihydrocollidine (DDC).

3,5-diethoxycarbonyl-1,4-dihydrocollidine (DDC)–induced liver injury is a well-established mouse model to study formation of Mallory bodies in various liver diseases and the mechanisms of oval cell activation and proliferation [6]. Furthermore, this model is used to investigate the mechanism of xenobiotic-induced cholangiopathies and shows pathological characteristics similar to that of human sclerosing cholangitis [7]. In addition, recent studies revealed that DDC-fed mice also show injuries in the heart, intestine, and hematopoietic organs [8–10]. However, no study has revealed whether renal impairment appears during the early phase of DDC feeding. We recently revealed that the morphological changes of bile canaliculi occur in the early phase of DDC feeding [7]. In the study, we concurrently observed the kidneys of DDC-fed mice using light microscopy but did not detect definitive morphological abnormalities. We assumed that DDC would induce modest damage in the kidney, and thus, these would be difficult to detect on routine examination.

Recent studies have revealed that cases of human AKI, including those involving patients with renal dysfunction associated with CLDs, often show almost normal tubules rather than necrotic tubules, even when the patient’s functional tests fulfill the diagnostic criteria of AKI [2, 11, 12]. Limited tubular alteration in the pathological findings of human AKI is known as sublethal tubular cell injury (sublethal injury hereafter), and it has become a subject of particular interest because of its contrast with the severe morphological changes seen in the tubules of animal AKI models [12].

The term *sublethal injury* indicates any type of damage short of cell death in which the cell is still viable, where both functional and morphological changes may occur [12]. The injury is often located on the border between necrotic and unaffected cells in various organs (e.g., the stunned myocardium of the heart in patients with ischemic heart diseases and the penumbra of the brain in patients with cerebral infarction) [13, 14]. In the kidney, Heyman et al. reviewed previous reports describing morphological changes in sublethal injury and summarized that there are very minor pathological changes that are almost undetectable by light microscopy with routine staining; only electron microscopy can reveal loss of brush borders, mitochondrial deformities, cytoplasmic vacuolization, or nuclear pyknosis [12]. The mechanism underlying sublethal injury has not been clarified, but mitochondrial injury is thought to be a primary mechanism [15–17].

Herein we describe that sublethal injury with mitochondrial damage and oxidative stress is detectable in the kidneys of DDC-fed mice from the early phase of feeding. A CLD mouse model showing AKI with sublethal injury would be useful for further study of the pathophysiological mechanisms of renal impairment in patients with CLDs and for testing novel therapeutic and protective strategies.

## Materials and Methods

### Animals

Eight-week-old male BALB/c mice (Japan SLC, Shizuoka, Japan) were used for all experiments, and age-matched BALB/c mice served as control subjects. To study DDC effects on the kidneys in the early phase, mice were fed a diet containing 0.1% DDC (Sigma-Aldrich, St. Louis, MO, USA) for 1, 3, and 7 days. The mice were housed in cages under specific pathogen-free conditions with a 12-hour light/dark cycle, and were provided food and water *ad libitum*. The control mice were fed a standard diet. To assess dose-response effects of DDC feeding, the mice were fed a diet containing 0.5% DDC for 1, 3, and 7 days. For feed-withdrawal analyses, another group of animals was fed a diet containing 0.1% DDC for 3 days and allowed to recover on a standard diet for an additional 7 or 28 days. To study DDC effects on the kidneys in longitudinal experiments, another group of mice was fed a diet containing 0.1% DDC for 14 and 56 days.

Mice were euthanized at each indicated time point for the subsequent experiments. Arterial blood was collected by cardiac puncture. For light microscopic analyses, kidneys were harvested, fixed by 4% buffered formaldehyde, and cut perpendicularly to the renal capsule to contain the cortex and medulla in a section. The liver samples were also harvested and fixed. For further morphological assessment, we performed transmission electron microscopy (TEM) using semi-thin sections with toluidine blue staining [7]. Briefly, the mice were perfused for 25 seconds with phosphate-buffered saline (PBS) and fixed by transcardial perfusion (~100 mmHg pressure) with 4% paraformaldehyde/2% glutaraldehyde. The kidneys were immersed in the same fixation solution at 4°C overnight. All experiments used five or more mice/group.

All animal experiments were approved by the Animal Care and Use Committee of Kyoto University, according to criteria outlined in the Guide for the Care and Use of Laboratory Animals prepared by the National Academy of Sciences, as published by the National Institutes of Health [18].

### Renal function analyses

Arterial blood gas analysis was immediately performed with an i-STAT 1 analyzer (Fuso Pharmaceutical Industries, Osaka, Japan). Subsequently, the remaining samples were centrifuged, and plasma samples were stored at -80°C prior to analysis of aspartate aminotransferase (AST), alanine aminotransferase (ALT), alkaline phosphatase (ALP), total bilirubin, and creatinine with a Hitachi 7180 analyzer (Hitachi, Tokyo, Japan); blood urea nitrogen (BUN) was analyzed with an i-STAT 1 analyzer. Urinary neutrophil gelatinase-associated lipocalin (NGAL) (Quantikine ELISA NGAL; R&D Systems, Minneapolis, MN, USA), urinary albumin (Albuwell M; Exocell, Philadelphia, PA, USA), and urinary creatinine (The Creatinine Companion; Exocell) were assayed according to the manufacturers’ instructions.

### Histological examinations

Formalin-fixed, paraffin-embedded tissue samples were cut in 3-μm-thick sections, deparaffinized, and stained with hematoxylin and eosin (H&E), periodic acid-Schiff (PAS), Azan, and reticulin staining. Other harvested samples were immediately frozen at the optimal cutting-temperature compound and stored at -80°C for further analyses.

To evaluate the grade of kidney histological changes, the following morphological parameters were evaluated for at least five randomly selected cortex and outer medulla areas at 200× magnification: proximal necrotic cells, brush border loss, cast formation, tubular dilatation, and neutrophil infiltration. The parameters were graded as follows: none (0); 1%–25% (1); ≥ 26% for parameters other than neutrophil infiltration (2); and none (0); 1–4 cells/field (1); ≥ 5 cells/field for neutrophil infiltration (2). The average of the sum of each parameter was calculated as the acute tubular necrosis (ATN) score.

The grading and staging of liver histological changes were evaluated as described previously [7]. Briefly, quantification of spotty necrotic cells was performed at 400× magnification, and grading of portal inflammation and periportal interface hepatitis and staging of fibrosis were performed according to the modified Ishak system at 200× magnification in at least five randomly selected areas. Mitotic cells were counted in the proximal tubules at 400× magnification, and the counts were expressed as average counts in a field. Histological changes were independently assessed by an experienced pathologist (TI) and an attending pathologist (HK) in a blinded fashion, and a consensus diagnosis was obtained for each sample.

### Immunohistochemistry

Immunohistochemical staining of the paraffin-embedded specimens was performed according to the manufacturer’s instructions. Briefly, specimens were deparaffinized, and antigen retrieval was carried out in a pressure cooker by boiling in 10 mM citrate buffer (pH 6.0), followed by rinsing with PBS. Subsequently, endogenous peroxidase was quenched with 3% H_2_O_2_ for 10 min at room temperature. After rinsing and the blocking process by 1% bovine serum albumin and 0.1% cold water fish-skin gelatin for 30 min at room temperature, slides were treated with negative control reagents or the following optimally diluted primary antibodies: anti-Ly-6G/-6C (rat monoclonal, 1:50; Abcam, Cambridge, MA, USA), anti-leukocyte common antigen (LCA) (mouse monoclonal, 1:200; Dako, Glostrup, Denmark), anti-Ki67 (mouse monoclonal, 1:100; Novocastra, New Castle, UK), anti-4-hydroxy-2-nonenal (4-HNE) (rabbit polyclonal, 1:500; Merck Millipore, Darmstadt, Germany), and anti-3-nitrotyrosine (3-NT) (rabbit polyclonal, 1:100; Merck Millipore) for 1 hour at room temperature. After rinsing, the slides were incubated with anti-mouse or anti-rabbit horseradish peroxidase (HRP)–conjugated secondary antibody (goat polyclonal, prediluted; MBL, Aichi, Japan) for 30 min at room temperature. Diaminobenzidine (DAB Substrate Solution; MBL) was used as a chromogen, followed by counterstaining with hematoxylin.

For quantification of the regenerative ability of renal tubules and inflammation after DDC feeding, cells positive for Ki-67, Ly-6G/-6C, and LCA were counted, respectively, in at least five randomly selected cortex and outer medulla areas at 400× magnification (Ki-67) or 200× magnification (Ly-6G/-6C and LCA); counts were expressed as an average count at each magnification.

For quantification of oxidative stress, anti-4-HNE-positive areas were analyzed using ImageJ software (National Institutes of Health, Bethesda, MD, USA) in 10 randomly selected areas at 200× magnification, and 3-NT-positive cells were counted in five randomly selected cortex areas at 200× magnification. The counts in all epithelial cells were expressed as average percentages.

### Immunofluorescent analyses

Immunohistochemical staining of frozen sections was performed according to the manufacturer’s instructions. Briefly, frozen specimens were fixed by 4% paraformaldehyde for 30 min, cut to 6-μm thickness, blocked with 1% BSA, washed, and incubated with the following optimally diluted primary antibodies: anti-kidney injury molecule-1 (KIM-1) (rabbit polyclonal, 1:400; Abcam) or anti-p62 (guinea pig polyclonal, 1:800; Progen Pharmaceuticals, Heidelberg, Germany). Subsequently, slides were incubated with Alexa Fluor 488 goat anti-rabbit (Cell Signaling Technology, Danvers, MA, USA) and Alexa Fluor 488 goat anti-guinea pig (Jackson ImmunoResearch, West Grove, PA, USA) as the secondary antibody and Alexa Fluor 594 phalloidin and 4′,6-diamidino-2-phenylindole (DAPI) for nuclear staining (Thermo Fisher Scientific, Waltham, MA, USA).

For quantitative assessments of KIM-1 protein expression, the fluorescence images stained with anti-KIM-1 antibody were analyzed using ImageJ software in 10 randomly selected areas at 200× magnification.

### Electron microscopic analyses

To assess sublethal injuries in renal tubules, semi-thin sections and TEM images prepared from Epon-embedded tissues were used. Briefly, the kidneys were fixed with 4% paraformaldehyde/2% glutaraldehyde and cut into 1 mm^3^. The specimens were extensively washed with PBS, post-fixed in 1% osmium tetroxide, dehydrated in a graded series of ethanol, and embedded in Epon. Semi-thin sections (750-nm thickness) were stained with toluidine blue and examined; ultrathin sections (80-nm thickness) were stained with 1% uranyl acetate with lead citrate as counterstaining and examined on an H-7650 electron microscope (Hitachi).

For quantification of proximal tubular cell injury, damaged cells with shortened or lost brush border microvilli and all proximal tubular cells were counted in five randomly selected renal cortex areas in semi-thin sections stained with toluidine blue at 400× magnification. The damaged cells/total cells ratio was expressed as a percentage.

For the quantification of single-cell extrusion, eliminated renal proximal tubular cells and total renal proximal tubular cells were counted in five randomly selected areas at 500× magnification. The eliminated cells/total cells ratio was expressed as a percentage.

For quantification of autophagy, both autophagosomes and autolysosomes in renal proximal tubular cells were counted in five randomly selected areas at 1,000× magnification.

### Quantitative reverse transcription polymerase chain reaction

Quantitative reverse transcription polymerase chain reaction (RT-PCR) was performed as previously described [19]. Briefly, total RNA was extracted from kidney tissue using TRIzol (Invitrogen, Carlsbad, CA, USA). cDNA was synthesized from total RNA using the SuperScript III Reverse Transcriptase (Invitrogen). RT-PCR was performed using the FastStart SYBR Green Master (Roche Diagnostics, Basel, Switzerland) and Rotor-Gene Q (Qiagen, Venlo, The Netherlands). The primer pairs used are provided in S1 Table. Data analysis was performed using the 2−ΔΔCT method for relative quantification. The relative target gene expression was normalized to the Gapdh mRNA expression.

### Immunoblot analyses

For assessment of autophagy in the kidneys, immunoblot analysis of microtubule light-chain protein 3-phosphatidylethanolamine conjugate (LC3-II) was performed. Briefly, frozen kidney tissue samples were homogenized. Ten micrograms of the proteins were separated by sodium dodecyl sulfate-poly-acrylamide gel electrophoresis and transferred to polyvinylidene difluoride membranes. Subsequently, membranes were blocked with 2.5% nonfat milk/0.25% BSA overnight at 4°C and incubated with primary antibody of anti-LC3 antibody (1:1,000, rabbit polyclonal; Sigma-Aldrich, St. Louis, MO, USA) for 1 hour at 37°C. After washing in Tris-buffered saline, membranes were incubated with HRP-conjugated secondary antibody (1:5,000, goat polyclonal; Dako) for 30 min at 37°C. Proteins were detected with the Pierce Western blotting substrate plus kit (Thermo Fisher Scientific), visualized by LAS-4000 mini (Fujifilm, Tokyo, Japan), analyzed by gel densitometry using ImageJ software, and reported as a -fold change relative to control mice. Expression data were normalized to β-actin.

### Statistical analysis

Data are reported as arithmetic means ±SEM. Comparisons between groups were made using the two-tailed Student’s *t* test or the Mann–Whitney *U* test with Bonferroni correction for multiple comparisons using SPSS software, version 20 (IBM Japan, Tokyo, Japan). Statistical significance was defined as a *P* < 0.05.

## Results

### Renal dysfunction with tubular injuries in DDC-fed mice

We first assessed whether liver injuries occurred in DDC-fed mice. Functional tests and histological evaluations of the liver showed severe abnormalities as in previous reports (S1 Fig) [7].

**Fig 1:**
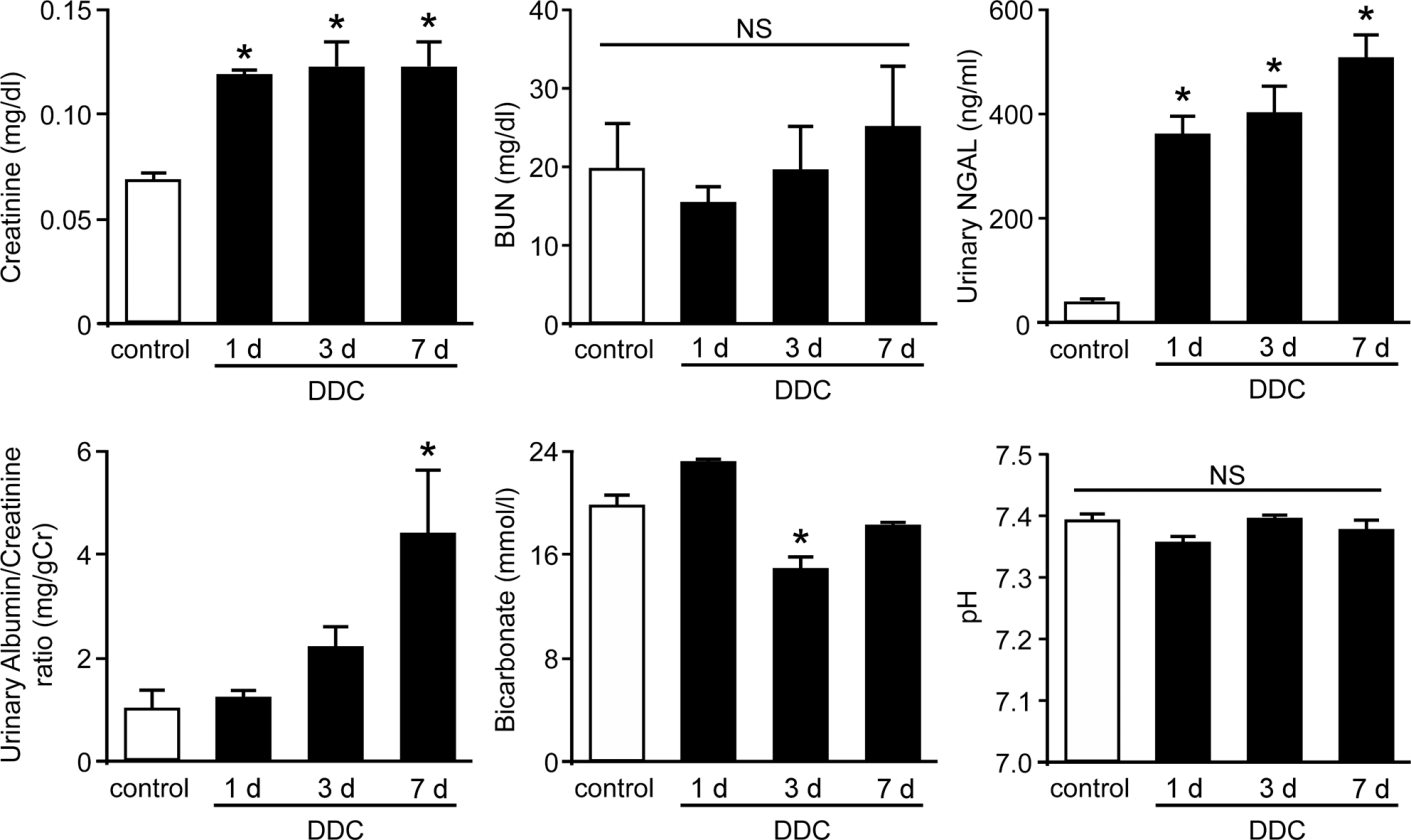
Renal dysfunction with tubular injuries in 3,5-diethoxycarbonyl-1,4-dihydrocollidine (DDC)-fed mice. Plasma creatinine levels, plasma blood urea nitrogen (BUN) levels, urinary neutrophil gelatinase-associated lipocalin (urinary NGAL) levels, urinary albumin/creatinine ratio, arterial blood bicarbonate, and arterial blood pH in control (*white bar*) and DDC-fed mice (*black bar*) at days 1, 3, and 7. Data are presented as means ± SEM. *P < 0.05 compared with control mice. NS, not significant.

To determine whether renal dysfunction occurs during the early phase of DDC-induced liver dysfunction, we obtained blood and urine samples from the DDC-fed mice after up to 7 days of feeding. We examined plasma creatinine and blood urea nitrogen levels for kidney function. The results showed that plasma creatinine levels were slightly but significantly increased from day 1 of DDC feeding compared to those of the control mice; the increase lasted throughout the early phase (Fig 1). Urinary examinations showed a high level of a tubular damage marker, urinary NGAL, and the urinary albumin/creatinine ratio were increased at day 7. In addition, arterial blood gas measurements showed that levels of bicarbonate concentration, a tubular epithelial damage marker, significantly decreased at day 3, but this was not associated with acidosis, as demonstrated by pH measurement. These results suggest that slight but definitive renal dysfunction associated with tubular injuries appears from the early phase of DDC-induced liver dysfunction.

### Kidneys of DDC-fed mice do not show definitive morphological changes on routine histological examination

**Fig 2:**
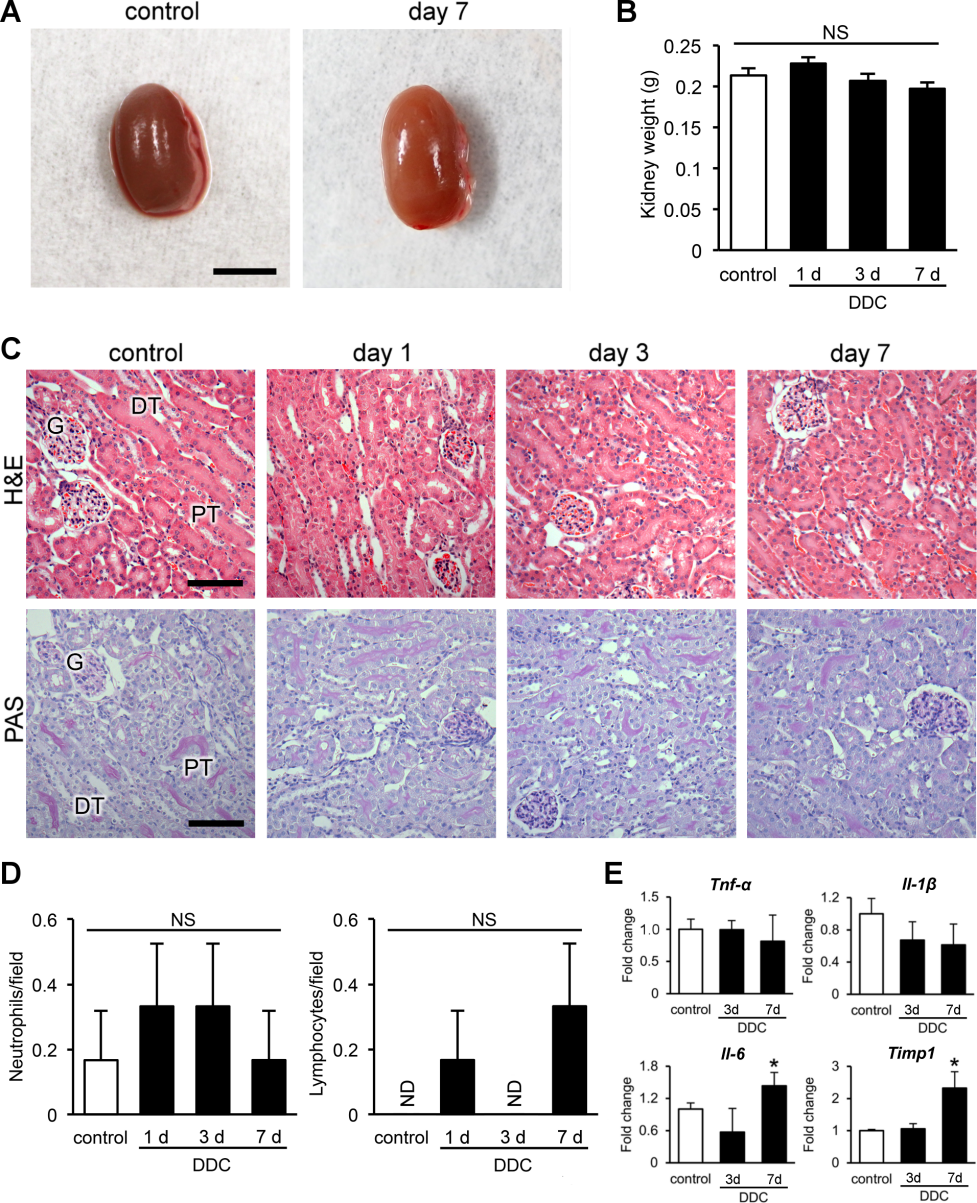
Kidneys of 3,5-diethoxycarbonyl-1,4-dihydrocollidine (DDC)-fed mice do not show definitive morphological changes on routine histological examination. (**A**) Representative macroscopic photographs of the kidneys. Scale bar = 5 mm. (**B**) Kidney weights in control (*white bar*) and DDC-fed mice (*black bar*) at days 1, 3, and 7. (**C**) Histopathological images of kidneys of control and DDC-fed mice at days 1, 3, and 7 (top row, hematoxylin and eosin [H&E] staining; bottom row, periodic acid-Schiff [PAS] staining). PT, proximal tubules; DT, distal tubules; G, glomeruli. Scale bars = 100 μm. (**D**) Counts of Ly-6G/-6C-positive neutrophils and leukocyte common antigen (LCA)–positive lymphocytes in control (*white bar*) and DDC-fed mice (*black bar*) at days 1, 3, and 7. Data are presented as means ± SEM. NS, not significant; ND, not detected. (**E**) Quantitative reverse transcription polymerase chain reaction analyses for tumor necrosis factor-α (*Tnf-α*), interleukin-1β (*Il-1β*), interleukin-6 (*Il-6*), and tissue inhibitor of metalloproteinase 1 (*Timp1*) mRNA in control and DDC-fed mice at days 3 and 7. Values are normalized to *Gapdh* expression. The mRNA values were analyzed by the two-tailed Student’s *t* test. Data are presented as means ± SD. *P < 0.05 compared with control mice.

Based on the results of the renal function analyses, we performed macroscopic and routine histological examinations of kidneys after up to 7 days of DDC feeding. In contrast to the results of the renal function analyses, the kidneys had no apparent macroscopic abnormalities, including kidney weights, throughout the period (Fig 2A and 2B). Histologically, H&E and PAS staining, the most common staining methods for renal histology, also showed no obvious morphological changes in the kidneys (Fig 2C). Tubular cell necrosis, apoptosis, and inflammatory changes were not observed in either cortical or medullary areas; ATN scores were 0 (Table 1). Immunohistochemistry for neutrophils and lymphocytes showed no significant difference in all groups of DDC-fed mice compared to control mice (Fig 2D and S2 Fig). Furthermore, apoptotic tubular cells were not observed in cortical or medullary areas, and the terminal deoxynucleotidyl transferase dUTP nick end labeling (TUNEL) assay and immunohistochemistry for active caspase-3 did not show apoptosis (S2 Fig).

**Table 1:** Morphological changes of the kidney and liver in DDC-fed mice.

		Control	Day 1		Day 3		Day 7	
			0.1%	0.5%	0.1%	0.5%	0.1%	0.5%
Kidney	ATN score	0.0 ± 0.0	0.0 ± 0.0	0.2 ± 0.2	0.0 ± 0.0	0.0 ± 0.0	0.0 ± 0.0	0.2 ± 0.2
Liver	Modified Ishak score							
	Necrotic cells	0.2 ± 0.1	10 ± 0.3	16 ± 1.0*	8.8 ± 1.4	9.3 ± 0.8	2.0 ± 0.2	2.3 ± 0.6
	Portal inflammation	0.2 ± 0.2	1.0 ± 0.0	1.0 ± 0.0	1.0 ± 0.0	1.5 ± 0.2	2.3 ± 0.2	2.0 ± 0.0
	Interface hepatitis	0.0 ± 0.0	0.3 ± 0.2	0.5 ± 0.2	1.0 ± 0.0	1.3 ± 0.2	2.8 ± 0.2	3.7 ± 0.2
	Fibrosis	0.0 ± 0.0	0.0 ± 0.0	0.0 ± 0.0	0.0 ± 0.0	0.0 ± 0.0	1.0 ± 0.0	1.0 ± 0.0

Acute tubular necrosis (ATN) scores and modified Ishak scores in control and 0.1% and 0.5% DDC-fed mice after 1, 3, and 7 days. Data are presented as mean ± SEM. DDC, 3,5-diethoxycarbonyl-1,4-dihydrocollidine. *P < 0.05 compared with the 0.1% DDC-fed mice.

To further understand the inflammatory responses induced by DDC, we next examined the gene expression changes in the kidneys of DDC-fed mice by quantitative RT-PCR analyses. The mRNA levels of proinflammatory cytokines (*Tnf-α* and *Il-1β*) showed no change in the early phase of DDC feeding, except for *Il-6* at day 7 (Fig 2E). Meanwhile, mRNA levels of a fibrogenic cytokine, *Timp1*, increased significantly at day 7, indicating that fibroblast activation emerged at the transcriptional level without apparent morphological inflammatory responses toward the end of the early phase. The data suggest that DDC does not induce apparent morphological changes in kidneys on routine light microscopy, despite the appearance of renal dysfunction and the expression changes of inflammation- and fibrosis-related genes.

### Sublethal tubular cell injuries are detectable in Epon-embedded kidney tissue samples from DDC-fed mice

We hypothesized that DDC feeding would lead to sublethal injuries of kidneys, because routine histological examinations did not show definitive morphological changes regardless of the functional damage. Sublethal injuries usually show limited morphological abnormalities detectable only by electron microscopy [12, 16]. Therefore, we next performed histological evaluation by toluidine blue examination using semi-thin sections (750-nm thickness) and transmission electron microscopic (TEM) analyses using ultrathin sections (80-nm thickness). The sections were prepared from perfusion-fixed and Epon-embedded kidney tissue samples because those sections provide finer images than do formaldehyde-fixed ones. Glomeruli, non-proximal renal tubules, and endothelia of DDC-fed mice did not show apparent morphological changes in the TEM images (S3 Fig).

**Fig 3:**
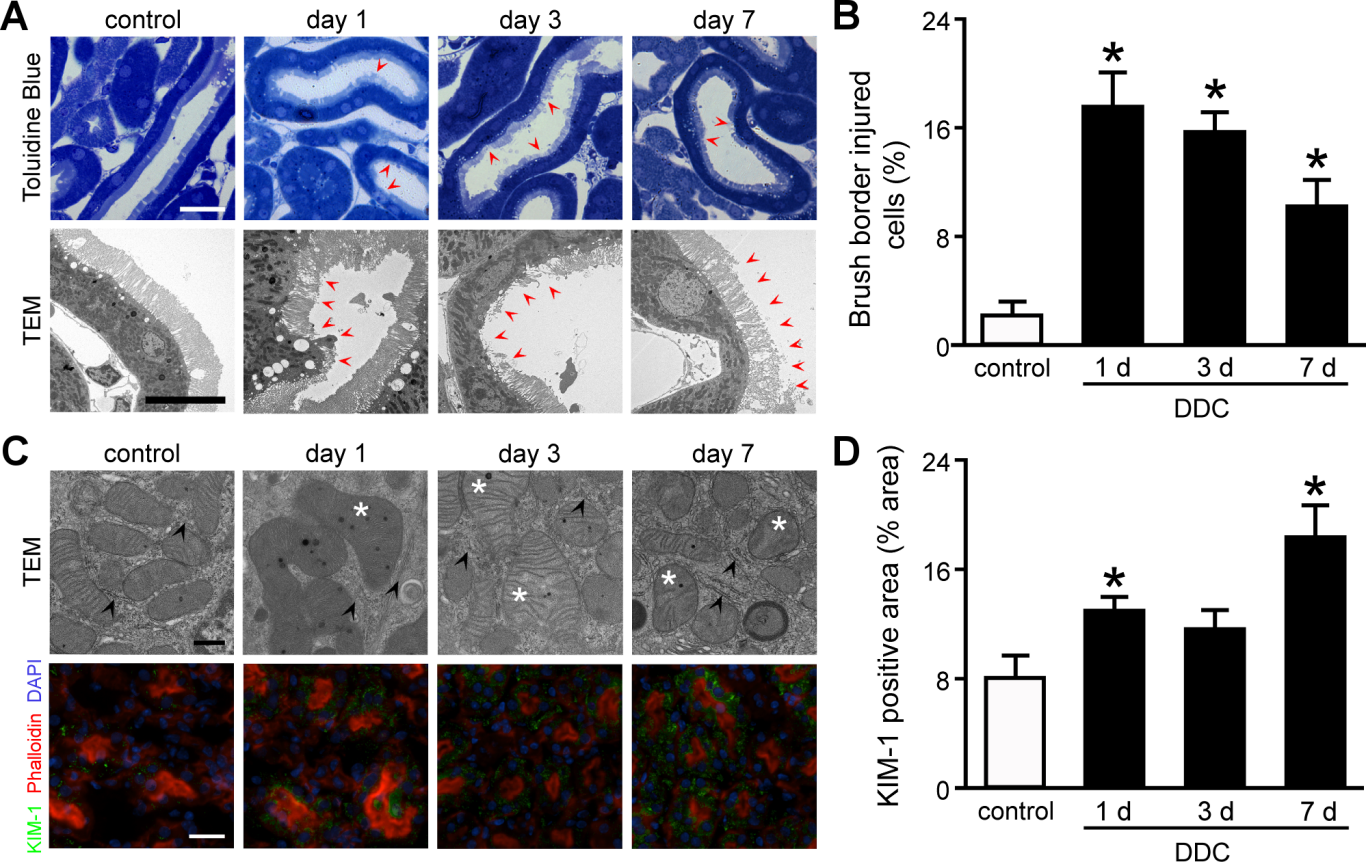
Sublethal tubular cell injuries in the kidney of 3,5-diethoxycarbonyl-1,4-dihydrocollidine (DDC)-fed mice. (**A**) Semi-thin sections with toluidine blue staining (top row) and transmission electron microscopic (TEM) (bottom row) images of proximal tubules in control and DDC-fed mice at days 1, 3, and 7; *red arrowheads*, loss of brush border microvilli. Scale bars = 25 μm (top row), 10 μm (bottom row). (**B**) The proportion of injured cells to total tubular cells is presented as percentage; control (*white bar*) and DDC-fed mice (*black bar*) at days 1, 3, and 7. (**C**) TEM images of intracellular organelles (top row) and immunofluorescent analysis for KIM-1 (bottom row) in proximal tubules of control and DDC-fed mice at days 1, 3, and 7; *asterisks*, mitochondria with abnormal features; *arrowheads*, intact endoplasmic reticula; *green*, immunofluorescence labeling for KIM-1; *red*, phalloidin; *blue*, 4,6-diamidino-2-phenylindole (DAPI). Scale bars = 500 nm (top row), 50 μm (bottom row). (**D**) KIM-1 staining on renal tubules of the control and DDC-fed mice was digitally analyzed and presented as percentage positive staining area per field; control (*white bar*) and DDC-fed mice (*black bar*) at days 1, 3, and 7. Data are presented as means ± SEM. *P < 0.05 compared with control mice.

Proximal tubular cells in the semi-thin sections and TEM analyses showed loss of brush border microvilli from day 1 of DDC feeding onward (Fig 3A). The brush border abnormalities were undetectable during routine histological examinations (Fig 2C). Quantitative assessment of semi-thin sections showed that the proportion of injured cells was significantly increased by 7.8-fold at day 1 of DDC feeding compared to that in the control mice (Fig 3B). Interestingly, the proportion of injured cells were decreased from days 1 to 7 in a time-dependent manner despite continued feeding, suggesting that the sublethal injury may have defense mechanisms against DDC intoxication [20].

Next, we investigated the intracellular organelles of proximal tubular cells by TEM and identified mitochondrial abnormalities: swelling, disarranged cristae, fusion, and fission. Meanwhile, other abnormalities, such as dilatation of endoplasmic reticulum (ER) and nuclear pyknosis, were not detected (Fig 3C), suggesting that DDC feeding led to mitochondrial damage rather than ER or nucleus damage.

For further evaluation of tubular cell injuries, we next assessed the expression of KIM-1, a well-known marker for tubular injuries, in kidneys from DDC-fed mice by immunofluorescent analysis. Consequently, KIM-1 expression was increased significantly in the cytoplasm of proximal tubular cells from day 1 of DDC feeding compared to that of control mice (Fig 3C). Quantitative densitometric analyses showed that the expression increased significantly by 1.6- and 2.3-fold over control mice at days 1 and 7, respectively (Fig 3D). These data suggest that sublethal injury with abnormalities of brush border microvilli and mitochondria appears in the kidneys of DDC-fed mice from the beginning of feeding.

### Single-cell extrusion with subsequent regeneration in the kidney of DDC-fed mice

**Fig 4:**
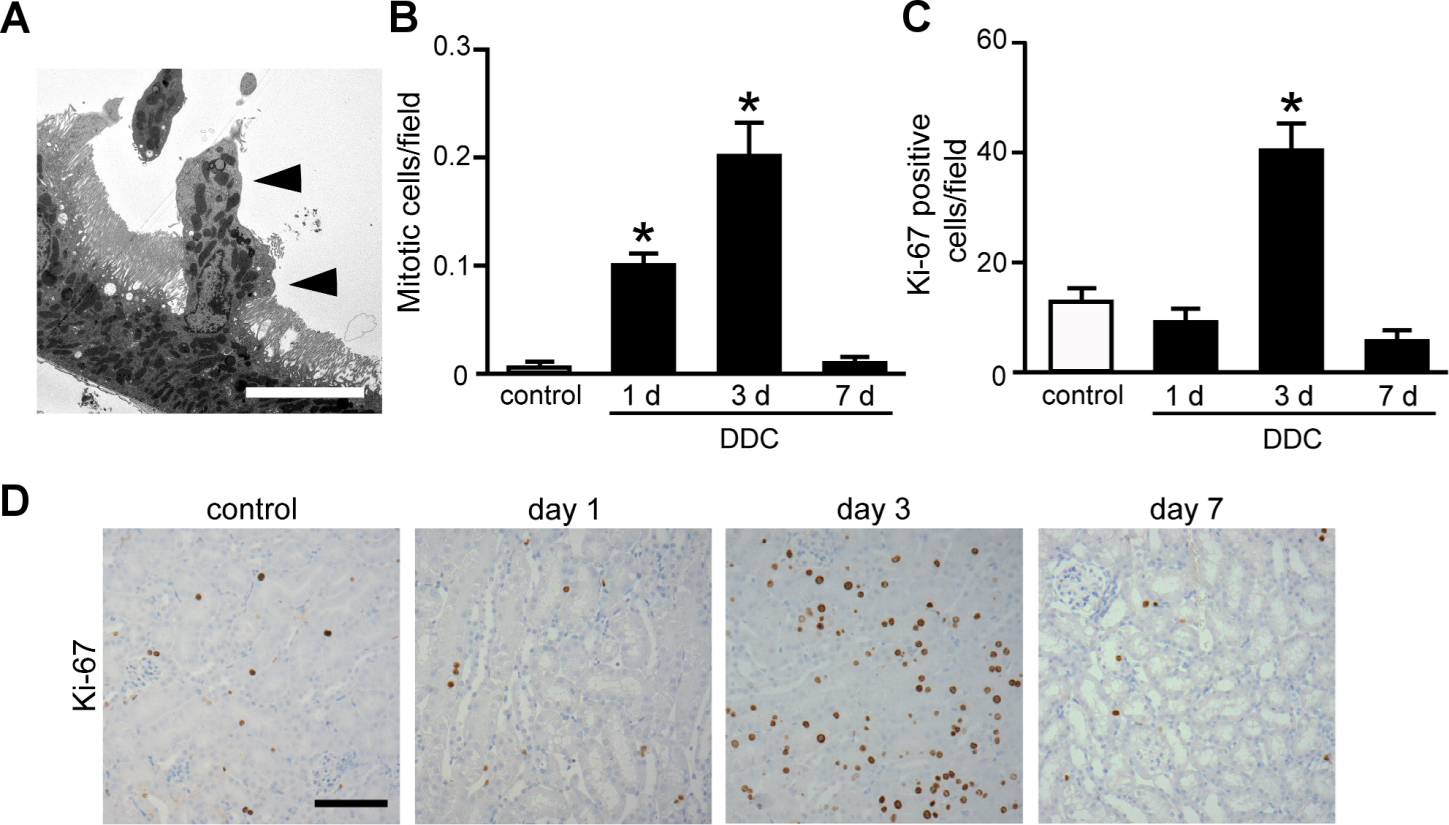
Single-cell extrusion with subsequent regeneration in the kidney of 3,5-diethoxycarbonyl-1,4-dihydrocollidine (DDC)-fed mice. (**A**) A representative photograph of a single tubular cell extrusion (*triangles*) from the epithelial layer of proximal tubules at day 3. Scale bar = 10 μm. (**B**) Counts of mitotic cells per field on proximal tubules of control (*white bar*) and DDC-fed mice (*black bar*) at days 1, 3, and 7. (**C**) Counts of Ki-67-positive cells per field on proximal tubules of control (*white bar*) and DDC-fed mice (*black bar*) at days 1, 3, and 7. Data are presented as means ± SEM. *P < 0.05 compared with control mice. (**D**) Immunohistochemical staining for Ki-67 in the proximal tubules of control and DDC-fed mice at days 1, 3, and 7. Scale bar = 100 μm.

Another noteworthy result was that TEM analysis revealed the extrusion of a single tubular cell from the epithelial layer of the proximal tubules at days 1 and 3 of feeding (Fig 4A). The eliminated cells were not associated with necrosis, apoptosis, or inflammatory cell infiltration. The proportion of eliminated cells among proximal tubular cells in DDC-fed mice at days 1 and 3 were 9% and 8%, respectively. Eliminated cells were undetectable in control and DDC-fed mice at day 7. We then examined whether tubular cell regeneration occurred subsequent to the cell extrusion. Histological examination showed a significant increase in mitotic cells in the proximal tubular cells at days 1 and 3 of DDC feeding compared to that in the control mice (Fig 4B and S4 Fig). Furthermore, immunostaining for a regeneration marker, Ki-67, revealed significantly increased positive cells at day 3 (Fig 4C and 4D). These data suggest that single-cell extrusion with subsequent regeneration makes the renal tubules appear to be intact and provide a defense mechanism against DDC toxicity.

### Mitochondrial damage and subsequent autophagy in the kidneys of DDC-fed mice

**Fig 5:**
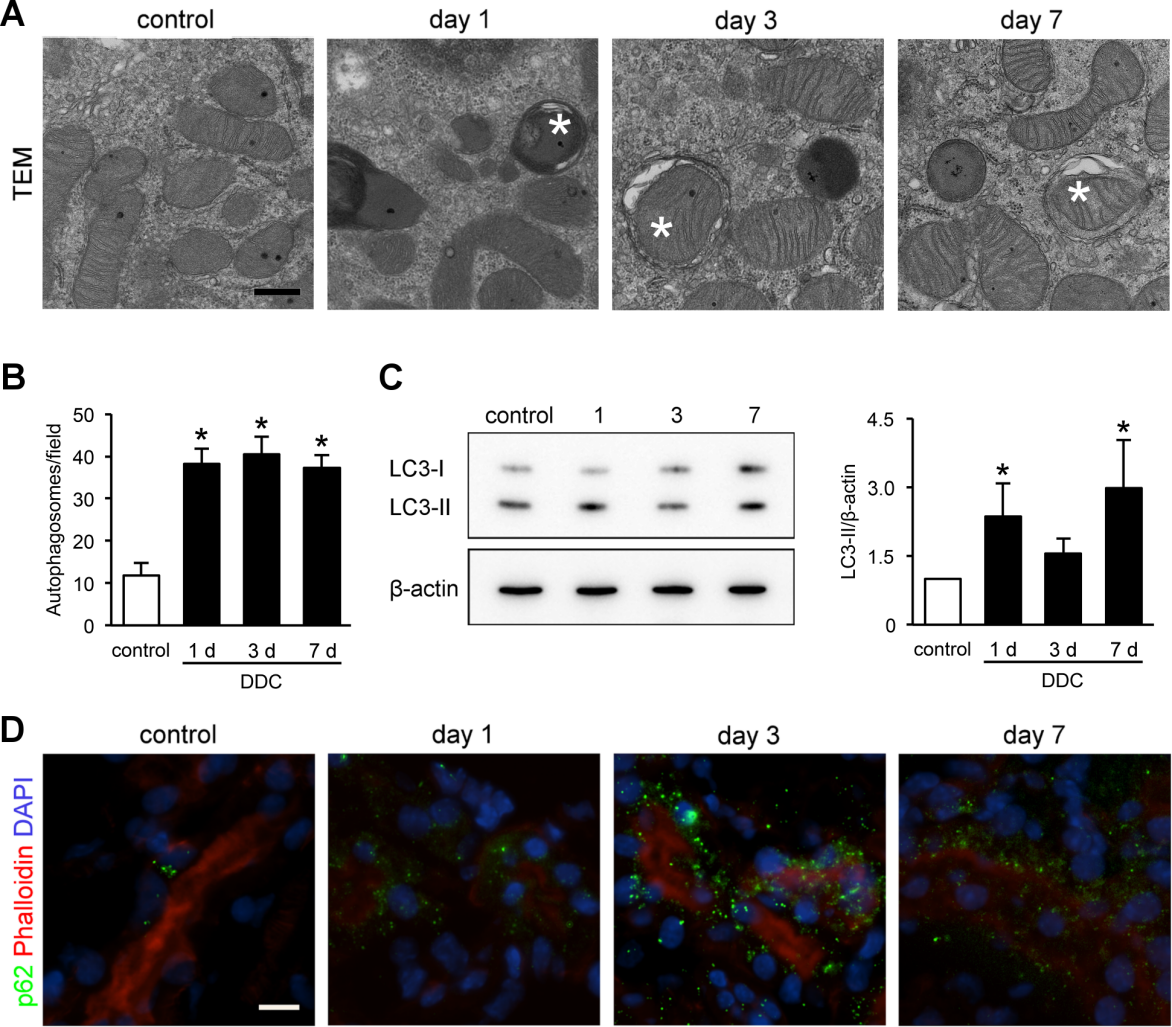
Mitochondrial damage and subsequent autophagy in the kidneys of 3,5-diethoxycarbonyl-1,4-dihydrocollidine (DDC)–fed mice. (**A**) Representative transmission electron microscopic images of autophagosomes in the proximal tubules of kidneys from control and DDC-fed mice at days 1, 3, and 7; *asterisks*, autophagosomes. Scale bar = 500 nm. (**B**) Counts of autophagosomes per 1,000× magnified field; control (*white bar*) and DDC-fed mice (*black bar*) at days 1, 3, and 7. (**C**) LC3-I and LC3-II protein expressions in the kidneys of control and DDC-fed mice at days 1, 3, and 7 by immunoblot analysis; β-actin served as a loading control; control (*white bar*) and DDC-fed mice (*black bar*) at days 1, 3, and 7. (**D**) Representative images of p62 immunostaining in kidneys of control and DDC-fed mice at days 1, 3, and 7; *green*, immunofluorescence labeling for p62; *red*, phalloidin; *blue*, diamidino-2-phenylindole (DAPI). Scale bar = 10 μm. Data are presented as means ± SEM. *P < 0.05 compared with control mice.

The kidneys of the DDC-fed mice exhibited mitochondrial injuries (Fig 3C). As injured mitochondria are engulfed by autophagosomes to maintain intracellular homeostasis in AKI, we hypothesized that autophagy also occurred in the injured proximal tubular cells of DDC-fed mice and prevented the injuries from progressing to cell death [16, 20]. We performed chronological TEM analyses and immunoblotting for microtubule LC3-I/II and immunofluorescence for p62 [21]. TEM analyses showed that autophagosomes increased significantly by 3.3-fold in the proximal tubular cells from day 1, and the number of autophagosomes remained high up to day 7 (Fig 5A and 5B). We also measured a reliable autophagy marker, LC3-II, in the kidney tissues [22]. The LC3-II/β-actin expression ratio was increased significantly at days 1 and 7, and the levels at day 3 tended to be increased (Fig 5C). LC3-II/LC3-I ratio showed an increasing tendency at day 3, but the levels did not reach statistical significance (DDC-fed mice day 1, 0.97 ± 0.20, *P* = 0.15, compared with control mice; day 3, 2.4 ± 0.55, *P* = 0.15; day 7, 1.3 ± 0.29, *P* = 0.69). Moreover, an immunofluorescent analysis for p62 showed a dot-pattern appearance in DDC-fed mice, indicating p62 accumulation on autophagosomes (Fig 5D). These results suggest that mitochondrial injuries in proximal tubular cells of kidneys in DDC-fed mice are associated with autophagy, a possible defense mechanism against renal impairment.

### Sublethal injuries in the kidneys of DDC-fed mice are associated with oxidative stress

**Fig 6:**
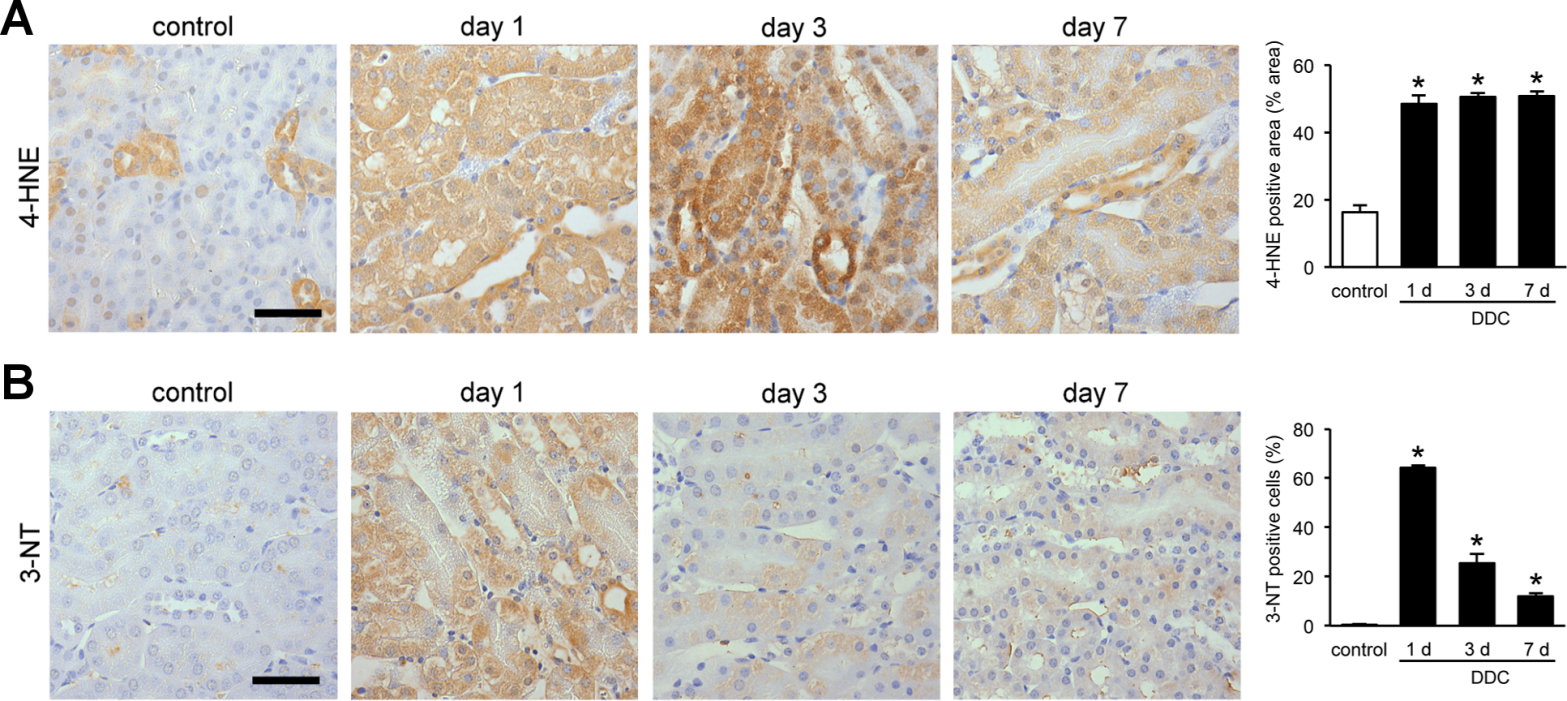
Sublethal injuries in the kidneys of 3,5-diethoxycarbonyl-1,4-dihydrocollidine (DDC)-fed mice are associated with oxidative stress. (**A**) Representative images of 4-hydroxy-2-nonenal (4-HNE) immunostaining in the kidneys of control and DDC-fed mice at days 1, 3, and 7. 4-HNE staining on kidney tissue was digitally analyzed and presented as percentage of positive staining area per field; control (*white bar*) and DDC-fed mice (*black bar*) at days 1, 3, and 7. Scale bar = 50 μm. (**B**) Representative images of 3-nitrotyrosine (3-NT) immunostaining in the kidneys of control and DDC-fed mice at days 1, 3, and 7. The ratio of 3-NT-positive cells to total tubular cells are presented as a percentage; control (*white bar*) and DDC-fed mice (*black bar*) at days 1, 3, and 7. Scale bar = 50 μm. Data are presented as means ± SEM. *P < 0.05 compared with control mice.

As mitochondrial injuries often lead to oxidative stress, we assessed whether DDC feeding induced oxidative stress in the renal tubules [23]. We performed immunohistochemical examination of 4-HNE and 3-NT, which are useful markers for oxidative stress in lipids and proteins, respectively [24]. 4-HNE expression in proximal tubules were increased significantly from day 1 and lasted throughout the early phase of DDC feeding (Fig 6A). The change in expression of distal tubules could not be assessed because of positive staining in the control mice. Meanwhile, 3-NT expression was increased in both the proximal and distal tubules from day 1, but gradually weakened over time (Fig 6B). The difference in the expression patterns between the two markers could be caused by the difference in injury mechanisms of oxidative stresses for lipids and proteins or the existence of different defense mechanisms [25, 26]. The data suggest that sublethal injuries in the kidneys of DDC-fed mice are associated with oxidative stress.

### Sublethal injury leads to interstitial fibrosis and fibroblast activation after long-term DDC feeding

**Fig 7:**
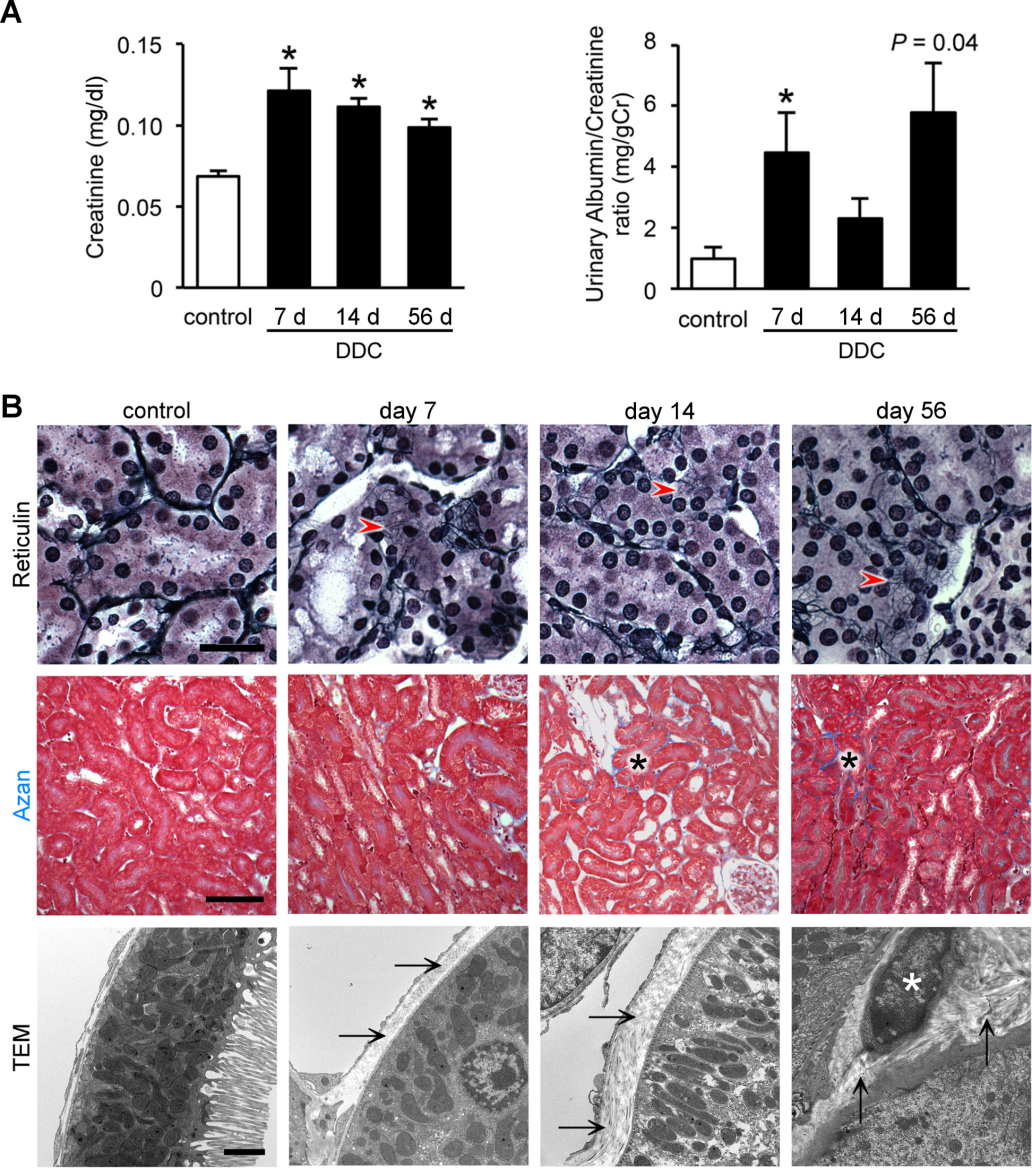
Sublethal injury leads to interstitial fibrosis and fibroblast activation after long-term 3,5-diethoxycarbonyl-1,4-dihydrocollidine (DDC) feeding. (**A**) Plasma creatinine levels and urinary albumin/creatinine ratio in control (*white bar*) and DDC-fed mice (*black bar*) at days 7, 14, and 56. Data are presented as means ± SEM. *P < 0.05 compared with the control mice. Control and 7 days of DDC-fed mice samples were reproduced from Fig 1; data were acquired using n ≥ 3 mice/group for the albumin/creatinine ratio. (**B**) Light and electron microscopic images of kidneys of control and DDC-fed mice at days 7, 14, and 56; top row, reticulin staining; middle row, Azan staining; bottom row, transmission electron microscopy (TEM); *red arrowheads*, accumulation of immature collagen fibers; *black asterisks*, accumulation of mature collagen fibers (blue staining); *arrows*, peritubular collagen fibers; *white asterisk*, myofibroblast with released collagen fibers. Scale bars = 25 μm (top row), 100 μm (middle row), and 2 μm (bottom row).

To evaluate progression of renal impairment in DDC-fed mice, we conducted a longitudinal evaluation of renal function and histology at days 7, 14, and 56. Pathological and functional tests of livers at day 56 of feeding showed a mild to moderate fibrosis, but no destruction of the lobular structure (S1 Fig). Before evaluating the effects of long-term DDC feeding on the kidney, we investigated whether aging influenced renal function in mice fed a standard diet. Consequently, 16-week-old mice (following an additional 56 days of standard-diet feeding) showed no significant deterioration of renal function or histology compared to 8-week-old mice (0 day, control mice) (data not shown), indicating that aging is a negligible factor in evaluating longitudinal experiments. Plasma creatinine concentrations at days 14 and 56 of DDC feeding showed significantly high levels, comparable to those of the control mice (Figs 1 and 7A). The urinary albumin/creatinine ratio also showed an increasing tendency at day 56, but the levels did not reach statistical significance (Figs 1 and 7A). Then, to evaluate whether these prolonged renal dysfunctions were associated with chronic histological changes, we performed Azan and reticulin staining to assess fibrotic changes in the kidneys of DDC-fed mice at 7, 14, and 56 days of the experimental diet. Reticulin stains immature collagen fibers (type III collagen) in black, forming a fine fluffy network. Azan stains mature collagen fibers (type I collagen) blue. Reticulin staining showed that accumulation of immature collagen fibers emerged around the tubules from day 7 (Fig 7B). Azan staining revealed that focal accumulation of mature collagen fibers appeared from day 14 and increased with time (Fig 7B). Furthermore, TEM analyses also showed that excessive peritubular collagen fibers appeared without inflammatory cell infiltration in a time-dependent manner, consistent with the results of the gene expression analyses (Fig 2E). Remarkably, active myofibroblast-releasing collagen fibers were found at day 56 (Fig 7B) [27]. Inflammatory cell infiltration or necrosis/apoptosis did not appear throughout the period. These data suggest that renal impairment of DDC-fed mice progresses to chronic kidney disease (CKD) in parallel with the progression of liver damage, with activated myofibroblasts, but is not associated with apparent inflammation. As activated myofibroblasts are closely associated with human AKI progression to CKD, the progression mechanism in DDC-fed mice may be similar to that in humans [27].

### The relationship between renal impairment and liver dysfunction in DDC-fed mice

**Fig 8:**
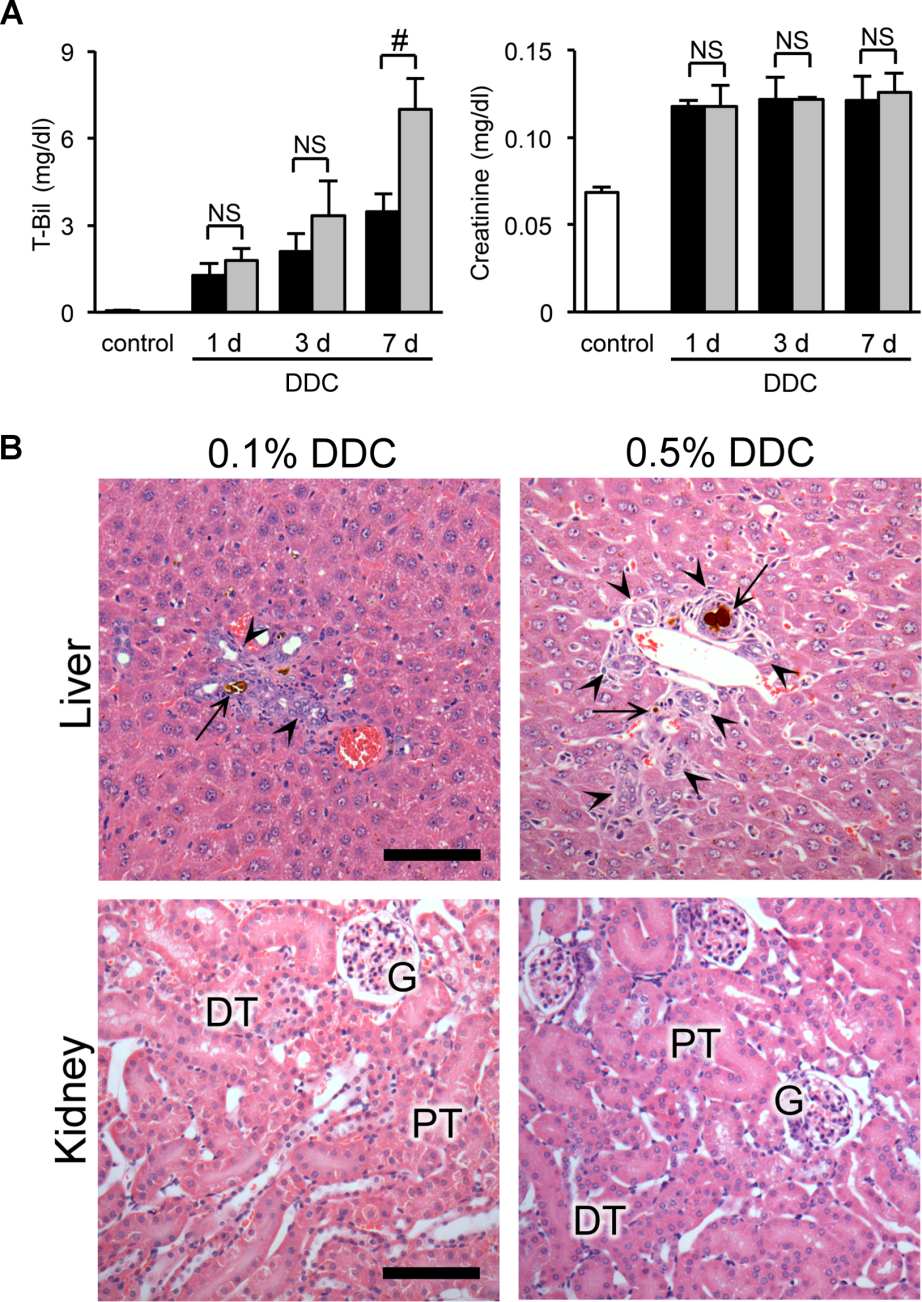
The relationship between renal impairment and liver dysfunction in dose-response analyses. (**A**) Serum levels of total bilirubin (T-Bil) and plasma creatinine in control (*white bar*), 0.1% DDC-fed (*black bar*), and 0.5% DDC-fed mice (*gray bar*) at days 1, 3, and 7. Data are presented as means ± SEM. ^#^*P* < 0.05 compared with the 0.1% DDC-fed mice. NS, not significant. Control and 0.1% DDC-fed mice samples were reproduced from Fig 1. (**B**) Representative photographs of livers and kidneys of 0.1% (left row) and 0.5% (right row) DDC-fed mice at day 7 (hematoxylin and eosin [H&E] staining). PT, proximal tubules; DT, distal tubules; G, glomeruli; *arrows*, porphyrin plugs; *arrowheads*, ductular reaction. Scale bars = 100 μm.

To further elucidate the relationship of renal impairment with liver dysfunction, we compared functional and morphological responses to DDC between the organs. We used two approaches: dose-response and feed-withdrawal analyses. In the dose-response analysis, serum levels of total bilirubin, a marker of liver injury, showed a significant increase in the 0.5% compared to the 0.1% diets at day 7 (Fig 8A). Meanwhile, plasma creatinine levels, a marker of kidney injury, did not show dose-dependent worsening throughout the early phase (Fig 8A). Histological examinations also showed aggravation of liver injuries in 0.5% DDC-fed mice, but no apparent deterioration in the kidneys (Fig 8B and Table 1).

**Fig 9:**
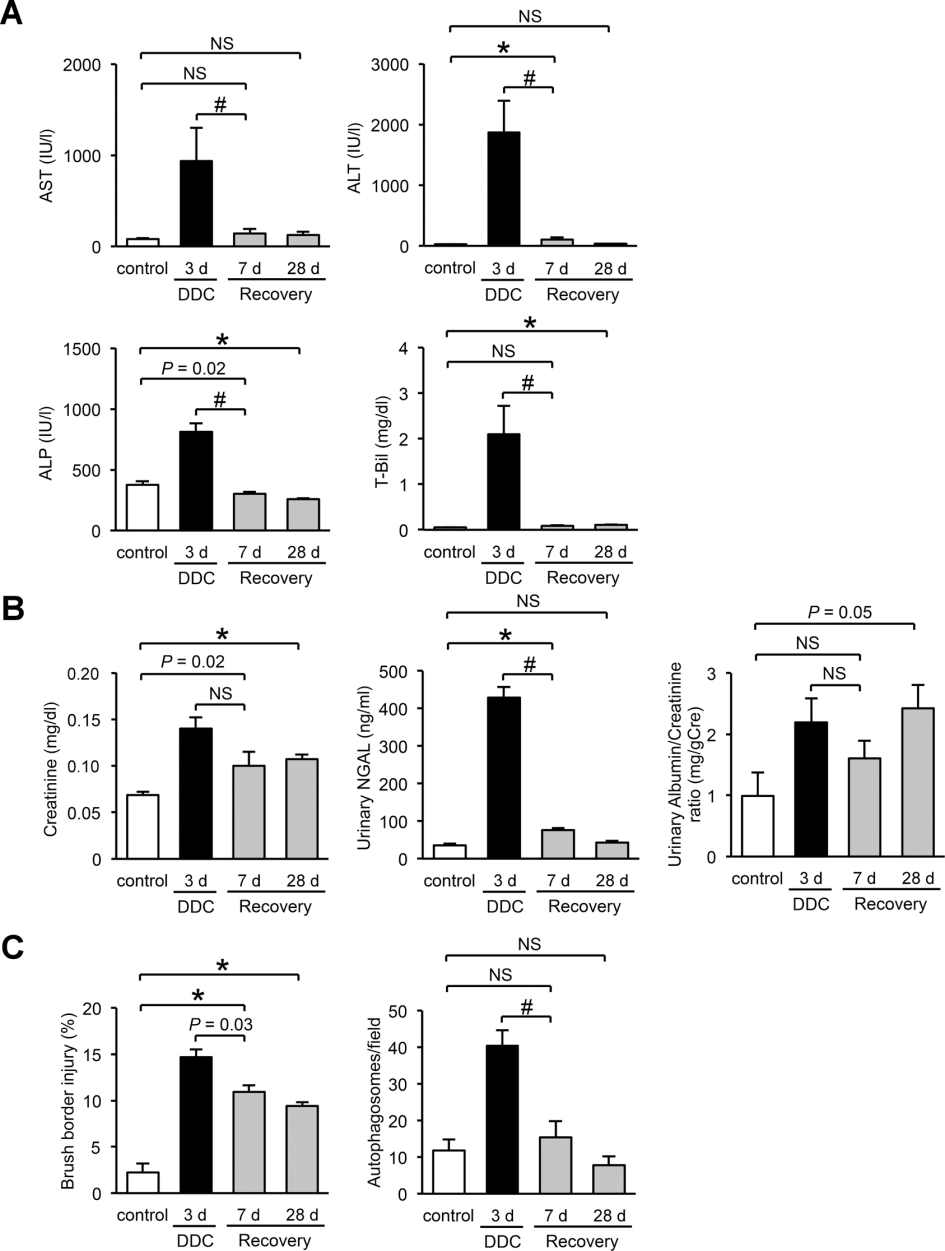
The relationship between renal impairment and liver dysfunction in feed-withdrawal analyses. (**A**) Serum levels of aspartate aminotransferase (AST), alanine aminotransferase (ALT), alkaline phosphate (ALP), and total bilirubin (T-Bil). (**B**) Serum levels of plasma creatinine, urine levels of urinary neutrophil gelatinase–associated lipocalin (NGAL), and urinary albumin/creatinine ratio. (**C**) Proximal tubular cells with injured brush border microvilli, and counts of autophagosomes in control (*white bar*), 3 days of DDC-fed (*black bar*), and recovery 7- or 28-day mice (3 days of DDC feeding followed by an additional 7 or 28 days of standard diet, *gray bar*). Data are presented as means ± SEM. *P < 0.05 compared with the control mice. ^#^*P* < 0.05 compared with three days of DDC-fed mice; NS, not significant. Control mice samples in creatinine, NGAL, and brush border injury, and control and 3 days of DDC-fed mice samples in T-Bil, urinary albumin/creatinine ratio, and autophagosomes were reproduced from Figs 1, 3, 5 and 8, respectively.

In the feed-withdrawal analysis, we fed mice DDC for 3 days and then returned them to a standard diet for an additional 7 or 28 days. Levels of renal and liver injury marker at day 3 of DDC feeding were significantly increased compared to those of the control mice (Fig 9A and 9B). Subsequently, 7 days on a standard diet caused an almost complete recovery of liver function (Fig 9A). In contrast, levels of renal injury markers showed a tendency to decrease but continued to be higher than those of control mice over an additional 28 days on the standard diet (Fig 9B). TEM analyses showed that injury to the brush border microvilli partially remained even after an additional 28 days on the standard diet, but autophagosome counts decreased to the levels of the control mice after 7 days of standard diet (Fig 9C). These data, including the former results of long-term feeding experiments, suggest that renal impairment in DDC-fed mice is impervious to the short-duration fluctuation of liver dysfunction, but this impairment progresses with the longitudinal advancement of CLD.

## Discussion

**Fig 10:**
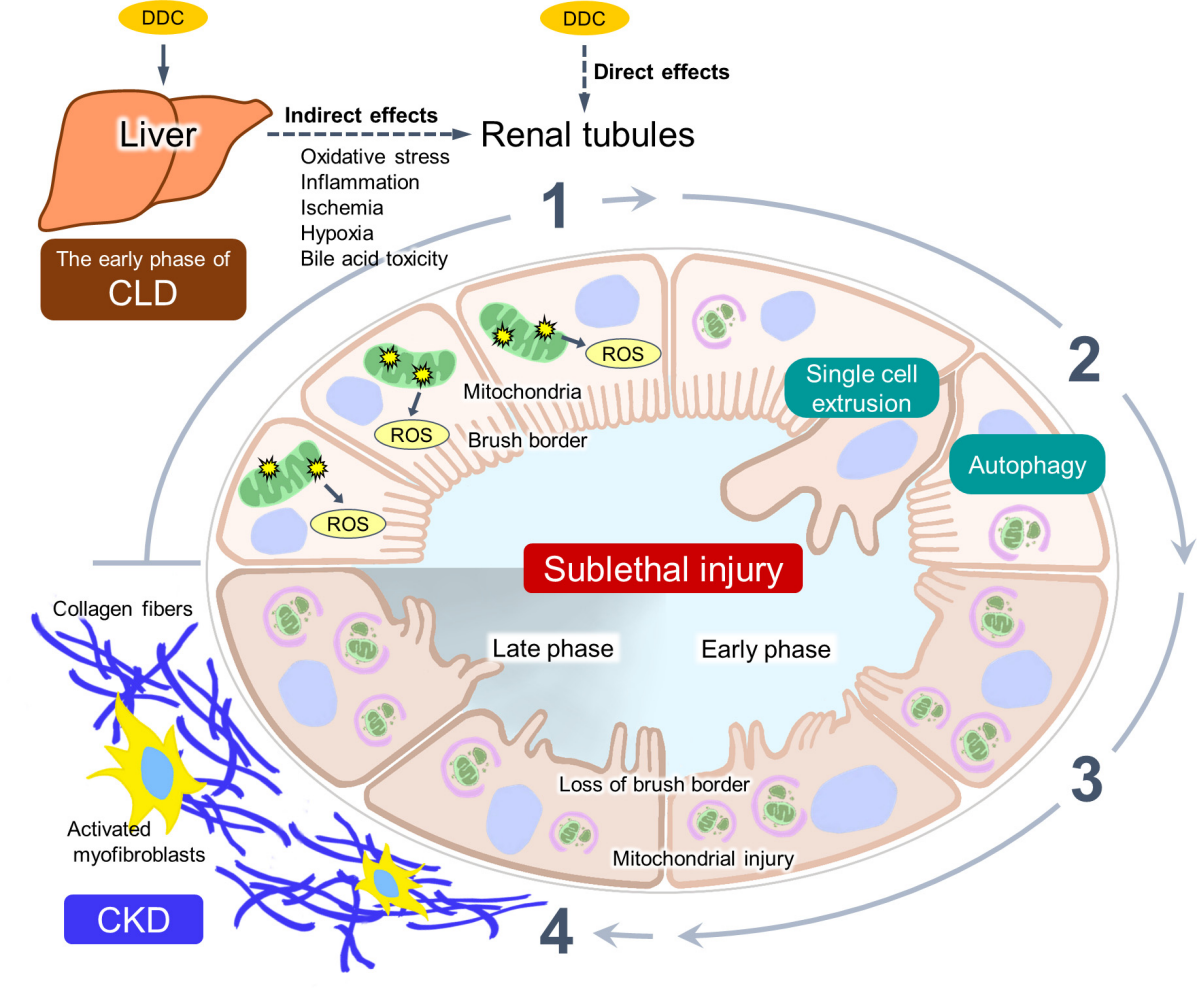
Proposed pathophysiology of renal impairment in 3,5-diethoxycarbonyl-1,4-dihydrocollidine (DDC)-induced chronic liver disease (CLD) model mice. DDC feeding leads to renal impairment with sublethal tubular cell injury. The tubules show: 1) the mitochondrial injuries and consequent oxidative stress with the generation of reactive oxygen species (ROS). 2) The injury is prevented from progressing to cell death through two defense mechanisms: autophagy and single-cell extrusion with regeneration. 3) The kidney remains in a state of sublethal injury with loss of brush border and mitochondrial injury (early phase). However, 4) activation and proliferation of myofibroblasts appear after long-term feeding (late phase), leading to an increased production of collagen fibers and progression to chronic kidney disease (CKD).

The major new finding of this study is that renal impairment with sublethal tubular cell injuries appears in DDC-fed mice from the beginning of feeding (Fig 10). Histological examinations of the kidneys with routine light microscopy did not show any apparent pathological findings in contrast to those of the liver, and electron microscopic analysis was required to detect morphological changes of sublethal injuries (i.e., brush border microvilli and mitochondrial abnormalities) [11, 12]. The functional and pathological damage was associated with mitochondrial injuries and oxidative stress, indicating the pathophysiological mechanism of sublethal injury involving the mitochondrial injury pathway. Intriguingly, this injury is proposed to have two defense mechanisms to keep it from progressing to apparent cell death: autophagy and a single-cell extrusion with regeneration. Furthermore, the sublethal injuries progressed to CKD with interstitial fibrosis in parallel with progression of liver dysfunction after long-term DDC feeding.

Our initial finding was that plasma creatinine levels of DDC-fed mice slightly but significantly increased compared to that in control mice [28]. This slight dysfunction is similar to that of patients who have normal creatinine levels but show progression to obvious AKI, called “subclinical AKI” [29]. Moreover, Epon semi-thin section analyses detected sublethal injuries. The semi-thin sections (750-nm thickness) with toluidine blue staining provided more definite images than did paraffin sections (3-μm thickness) because of strong fixation (paraformaldehyde, glutaraldehyde, and osmium tetroxide). In addition, semi-thin sections provide wider observation areas than does TEM. Thus, Epon sections are suitable for detecting sublethal injuries that show very slight and sporadically distributed changes, such as brush border microvilli abnormalities. Furthermore, we confirmed that the renal injury markers used in this study, urinary NGAL measurement, and KIM-1 imaging were highly sensitive. Epon sections and novel markers can be used to detect sublethal injuries, but the development of more affordable and widely available testing is needed for routine analyses.

Mitochondrial injury and oxidative stress are common mechanisms of cell injuries in a variety of human and animal AKI, including cisplatin-, ischemia-, or hypoxia-induced AKI [12, 15–17, 30, 31]. The current study revealed that mitochondrial injuries and oxidative stress occurred in the proximal tubular cells of DDC-fed mice. Oxidative stress generally occurs subsequent to mitochondrial injury, ER injury, or apoptosis [32, 33]. In the proximal tubular cells of DDC-fed mice, oxidative stress is thought to be induced by mitochondrial injuries, because only mitochondrial injuries but not ER injuries or apoptosis were detected in our model (Fig 3C and S2 Fig). Mitochondrial injuries followed by oxidative stress are the most likely mechanisms underlying sublethal injuries that appear in the kidneys of DDC-fed mice.

A sequence of mitochondrial injuries usually leads to additional damage in other organelles (DNA, plasma membrane, ER) via induction of reactive oxygen species, resulting in cell death [16, 17, 30], and longitudinal DDC feeding in this study continuously induced mitochondrial damage that led to CKD with interstitial fibrosis in parallel with CLD progression. In light of these results, we believe that DDC feeding led to cell death and was followed by inflammatory cell infiltration in the kidney. However, inflammatory cell infiltration or pronounced cell death did not occur during the study period. Instead, the results suggest that tubular cells have a unique defense mechanism against cell death, and consequently this study provides two possible mechanisms to prevent sublethal injuries from progressing to cell death: autophagy and single-cell extrusion with regeneration. Autophagy is a cell homeostatic mechanism; a recent study using genetic knockout mice suggests that autophagy blockade aggravates kidney injuries and the induction of autophagy promotes disease regression [20]. In our study, autophagosomes engulfing degraded mitochondria appeared from day 1 to day 56 (data not shown) of DDC feeding, suggesting that autophagy is a pivotal defense mechanism throughout the process to prevent progression to cell death that results in sustained sublethal injuries. Single-cell extrusion is another homeostatic mechanism used by epithelia to eliminate unnecessary or harmful cells from the epithelial layer [34]. In DDC-fed mice, extrusion and subsequent regeneration of tubular cells occurred only after up to three days of feeding. Therefore, this mechanism may protect an individual only in the very early phase of DDC feeding.

Sublethal injuries in the CLD mouse model could be induced not only by liver dysfunction but by direct effects of DDC on renal tubules, which may function separately or in collaboration. DDC could directly induce renal tubular injuries, including sublethal injuries, through mitochondrial dysfunction. DDC has been known to affect mitochondria through impaired heme biosynthesis because of excessive protoporphyrin IX accumulation due to ferrochelatase inhibition, and δ-aminolevulinate synthetase induction [35]. As heme deficiency leads to the impairment of the functions of various hemeproteins including mitochondrial electron transport chain-related proteins and cytochrome P450 [CYPs], DDC could affect renal proximal tubules with many mitochondria [16]. Meanwhile, sublethal injuries in the kidneys may be secondary to liver injuries through bile acids released from DDC-damaged liver tissues. Bile acids are released through bile duct obstruction by porphyrin plugs in DDC-induced liver injuries [6]. A recent study involving a liver transplantation rat model revealed that bile acids released from the injured liver acted as a mediator between early-phase injuries of the liver and kidney, and induced renal tubular injuries with mitochondrial abnormalities [5, 36]. Moreover, a bile duct ligation mouse model also demonstrated the negative effect of bile acids on the kidney [36]. This study illustrates that DDC feeding leads to sublethal injuries in the kidney; however, we did not elucidate the induction mechanism for these sublethal injuries. An *in vitro* DDC cytotoxicity assay using cultured renal epithelial cells may elucidate the relationship of DDC feeding with sublethal injury and address the limitations of our study.

In summary, this study demonstrates that renal impairment with sublethal tubular cell injuries appears in the DDC-induced CLD mouse model from the early phase. Mitochondrial injury and subsequent oxidative stress were associated with sublethal injuries, and autophagy and single-cell extrusion with regeneration may be defense mechanisms to prevent progression to apparent cell death. Furthermore, myofibroblasts appeared after long-term DDC feeding and released collagen fibers, leading to CKD, in parallel with hepatic injury progression. This mouse model, which shows sublethal tubular cell injury, could be useful for studying the pathophysiological mechanisms of renal impairment in the early phase of CLD.

## Supporting Information

S1 Fig Chronic liver injuries in DDC-fed mice. (**A**) Histopathological images of livers of control and DDC-fed mice on days 3 and 56 (top row, hematoxylin and eosin [H&E] staining; bottom row, Azan staining); C, central vein; P, portal vein; *broad arrows*, focal necrotic hepatocyte; *arrow heads*, ductular reaction; *narrow arrows*, porphyrin plugs; *asterisk*, fibrosis. Scale bars = 100 μm. (**B**) Serum levels of aspartate aminotransferase (AST), alanine aminotransferase (ALT), alkaline phosphate (ALP), and total bilirubin (T-Bil) in control (0-day, *white bar*) and DDC-fed mice at days 1, 3, 7, 14, and 56 (*black bar*). Control and 1, 3, and 7 days of DDC-fed mice samples in T-Bil data, and 3 days of DDC-fed mice samples in AST, ALT and ALP data were reproduced from Figs 7 and 8, respectively. (**C**) Modified Ishak system: counts of necrotic cells per field on liver tissue, histological grading of portal inflammation, histological grading of interface hepatitis, and histological staging of fibrosis in the controls (*white bar*) and DDC-fed mice (*black bar*). Data are presented as mean ± SEM; *P < 0.05 and **P < 0.01 compared with the controls. ND, not detected.
(PDF)

S2 Fig DDC feeding does not induce apparent inflammatory cell infiltration and apoptosis. (**A**) Representative photographs of immunohistochemistry for neutrophils (top row, *arrowheads*) and a lymphocyte (bottom row, *arrow*) for the detection of inflammatory cells in kidneys at day 1 of DDC feeding. Scale bars = 25 μm. (**B**) Representative photographs of terminal deoxynucleotidyl transferase dUTP nick end labeling (TUNEL) assay (top row) and immunohistochemistry for active caspase-3 (bottom row) in kidneys at day 3 of DDC feeding. Scale bars = 50 μm.
(PDF)

S3 Fig DDC does not induce pathological changes in glomeruli, distal tubules, and collecting ducts. Representative transmission electron microscopic images of the kidneys of control and DDC-fed mice at days 3 and 56; top row, glomeruli; middle row, distal tubules; bottom row, collecting ducts. Scale bars = 10 μm.
(PDF)

S4 Fig DDC induces mitoses of proximal tubular cells. A representative photograph of mitoses (*arrowheads*) in the proximal tubules of DDC-fed mice at day 3. Scale bar = 25 μm.
(PDF)

S1 Table RT-PCR primers for analysis. (PDF)
