# Supplementary figures and images for: Renal Impairment with Sublethal Tubular Cell Injury in a Chronic Liver Disease Mouse Model
Source: PLoS One. 2016 Jan 11;11(1):e0146871. doi: 10.1371/journal.pone.0146871 (PMC4713438; doi:10.1371/journal.pone.0146871)

S1 Fig. Chronic liver injuries in DDC-fed mice.

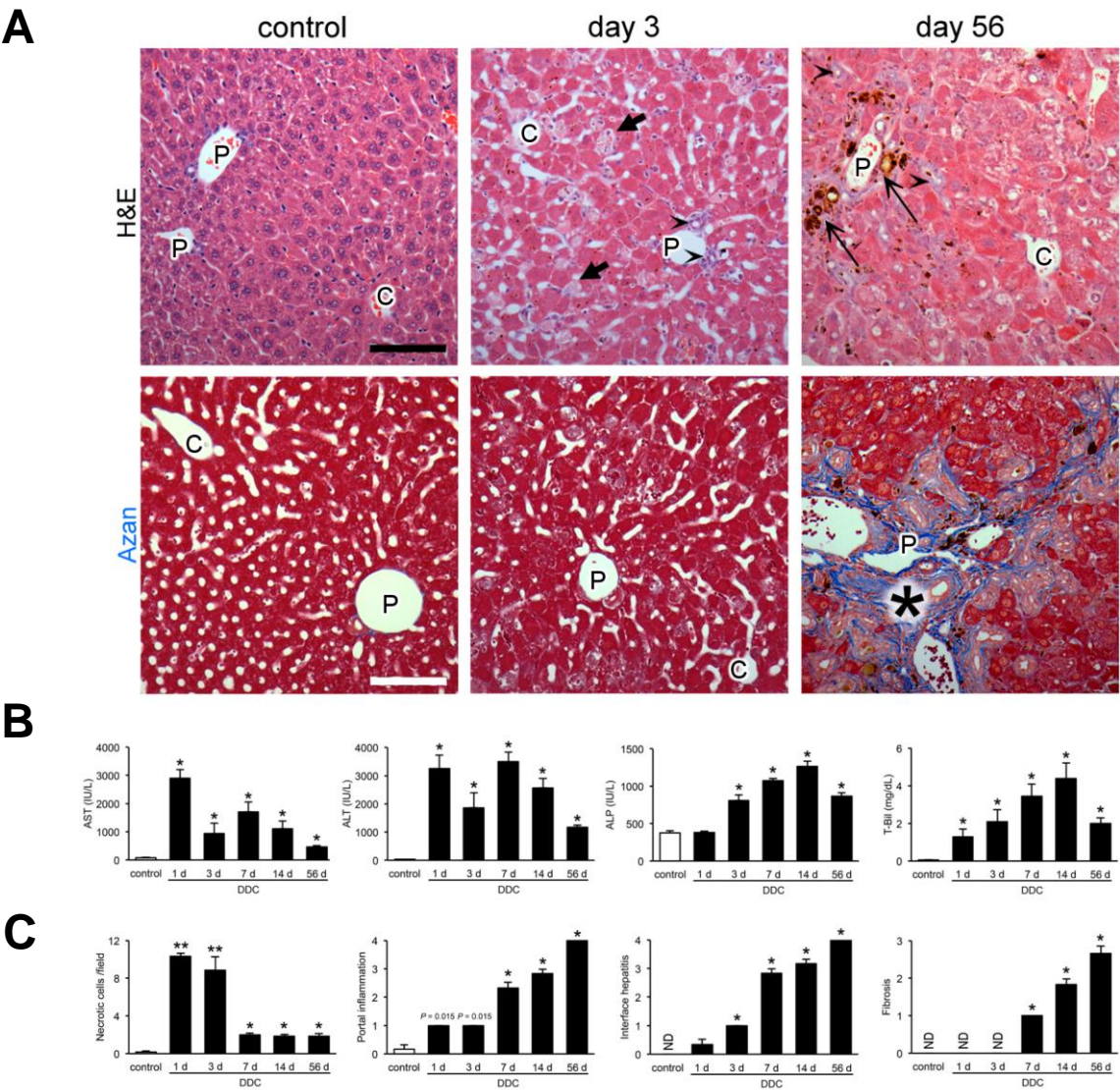

Supplement: S1 Fig — (A) Histopathological images of livers of control and DDC-fed mice on days 3 and 56 (top row, hematoxylin and eosin [H&E] staining; bottom row, Azan staining); C, central vein; P, portal vein; broad arrows, focal necrotic hepatocyte; arrow heads, ductular reaction; narrow arrows, porphyrin plugs; asterisk, fibrosis. Scale bars = 100 μm. (B) Serum levels of aspartate aminotransferase (AST), alanine aminotransferase (ALT), alkaline phosphate (ALP), and total bilirubin (T-Bil) in control (0-day, white bar) and DDC-fed mice at days 1, 3, 7, 14, and 56 (black bar). Control and 1, 3, and 7 days of DDC-fed mice samples in T-Bil data, and 3 days of DDC-fed mice samples in AST, ALT and ALP data were reproduced from Figs 7 and 8, respectively. (C) Modified Ishak system: counts of necrotic cells per field on liver tissue, histological grading of portal inflammation, histological grading of interface hepatitis, and histological staging of fibrosis in the controls (white bar) and DDC-fed mice (black bar). Data are presented as mean ± SEM; *P < 0.05 and **P < 0.01 compared with the controls. ND, not detected. (PDF) [file pone.0146871.s001.pdf]

S4 Fig. DDC induces mitoses of proximal tubular cells.

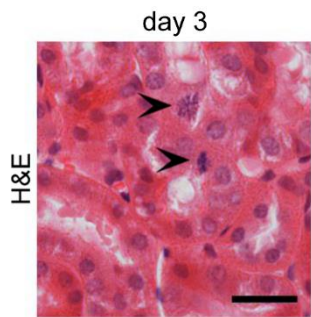

Supplement: S4 Fig — A representative photograph of mitoses (arrowheads) in the proximal tubules of DDC-fed mice at day 3. Scale bar = 25 μm. (PDF) [file pone.0146871.s004.pdf]
