# Supplementary material for: Renal Impairment with Sublethal Tubular Cell Injury in a Chronic Liver Disease Mouse Model
Source: PLoS One. 2016 Jan 11;11(1):e0146871. doi: 10.1371/journal.pone.0146871 (PMC4713438; doi:10.1371/journal.pone.0146871)

S3 Fig. DDC does not induce pathological changes in glomeruli, distal tubules, and collecting ducts.

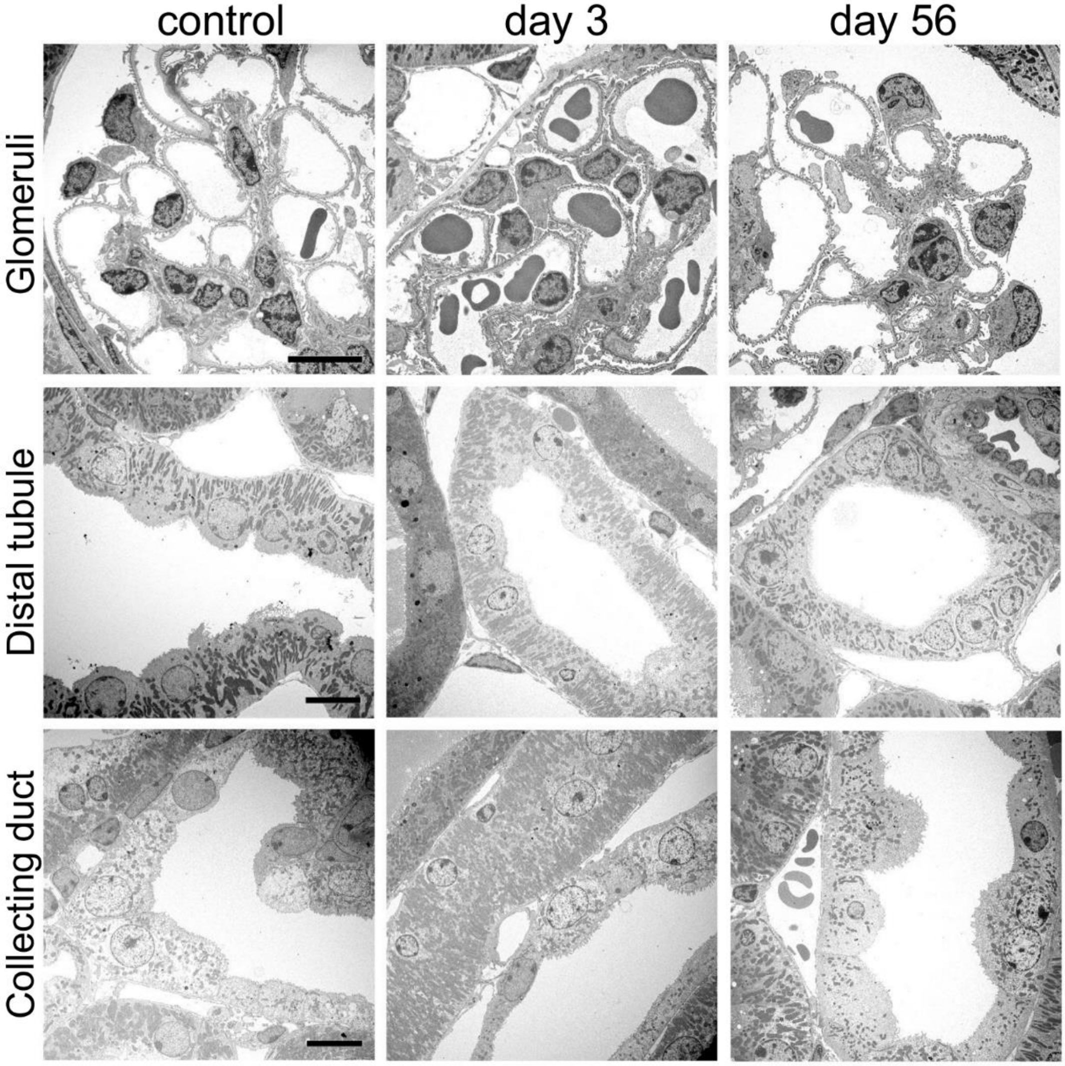

Supplement: S3 Fig — Representative transmission electron microscopic images of the kidneys of control and DDC-fed mice at days 3 and 56; top row, glomeruli; middle row, distal tubules; bottom row, collecting ducts. Scale bars = 10 μm. (PDF) [file pone.0146871.s003.pdf]
