# Supplementary material for: Renal Impairment with Sublethal Tubular Cell Injury in a Chronic Liver Disease Mouse Model
Source: PLoS One. 2016 Jan 11;11(1):e0146871. doi: 10.1371/journal.pone.0146871 (PMC4713438; doi:10.1371/journal.pone.0146871)
Supplement: S1 Table — (PDF) [file pone.0146871.s005.pdf]

S1 Table

RT-PCR primers for analysis

Gene	Direction	Sequence
Tnf-α	Forward	CCCTCACACTCAGATCATCTTCT
Tnf-α	Reverse	GCTACGACGTGGGCTACAG
Il-1β	Forward	CCAGCTTCAAATCTCACAGCAG
Il-1β	Reverse	CTTCTTTGGGTATTGCTTGGGATC
Il-6	Forward	TAGTCCTTCCTACCCCAATTTCC
Il-6	Reverse	TTGGTCCTTAGCCACTCCTTC
Timp1	Forward	GCCCTTCGCATGGACATTTA
Timp1	Reverse	CCCCGATCTGCGATGATG
Gapdh	Forward	AGGTCGGTGTGAACGGATTTG
Gapdh	Reverse	TGTAGACCATGTAGTTGAGGTCA

Tnf-α, tumor necrosis factor-α; *Il-1β*, interleukin-1β; *Il-6*, interleukin-6; *Timp1*, tissue inhibitor of metalloproteinase 1
