# Supplementary material for: Renal Impairment with Sublethal Tubular Cell Injury in a Chronic Liver Disease Mouse Model
Source: PLoS One. 2016 Jan 11;11(1):e0146871. doi: 10.1371/journal.pone.0146871 (PMC4713438; doi:10.1371/journal.pone.0146871)

S2 Fig. DDC-feeding does not induce apparent inflammatory cell infiltration and apoptosis.

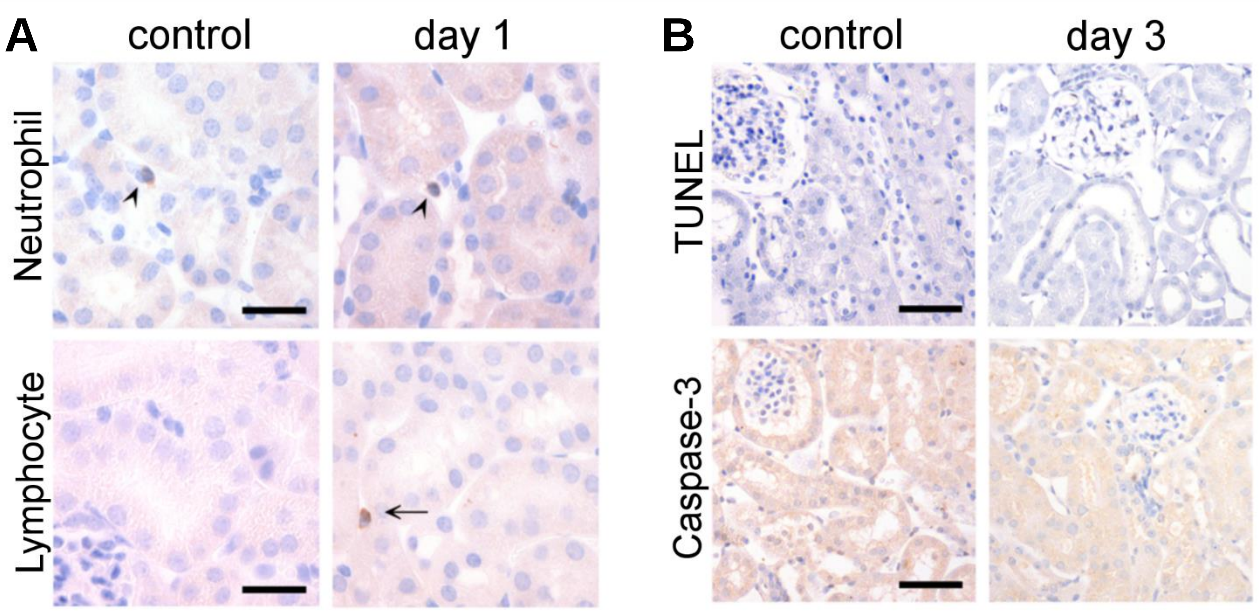

Supplement: S2 Fig — (A) Representative photographs of immunohistochemistry for neutrophils (top row, arrowheads) and a lymphocyte (bottom row, arrow) for the detection of inflammatory cells in kidneys at day 1 of DDC feeding. Scale bars = 25 μm. (B) Representative photographs of terminal deoxynucleotidyl transferase dUTP nick end labeling (TUNEL) assay (top row) and immunohistochemistry for active caspase-3 (bottom row) in kidneys at day 3 of DDC feeding. Scale bars = 50 μm. (PDF) [file pone.0146871.s002.pdf]
